# Supplementary material for: Relation of incident chronic disease with changes in muscle function, mobility, and self-reported health: Results from the Health and Retirement Study
Source: PLOS Glob Public Health. 2022 Sep 8;2(9):e0000283. doi: 10.1371/journal.pgph.0000283 (PMC10021154; doi:10.1371/journal.pgph.0000283)
Supplement: Attachment — Submitted filename: Responses to reviewers2.docx [file pgph.0000283.s001.docx]

**Response to Reviewers’ Comment on PGPH-D-21-00399**

**Reviewer #1’s Comments:** 
Introduction
● Explain what physical component score is

Response: The physical component score is a score from the SF-36 survey used in the referenced article. In the revision we briefly explain the physical component score: “Physical component score is based on eight scales of SF-36 and higher scores in physical component score indicates better physical health.” (Lines 64-65)

● A clear distinction needs to be made between the terms ‘comorbidities’ and ‘multimorbidities’-there is no mention of the term ‘comorbidities’

Response: To avoid the confusion, we deleted the word “comorbidities” or “multimorbidities” but consistently used “multiple chronic conditions” through the manuscript.

Methods
● It is unclear what is the study hypothesis is and how did authors test the hypothesis.

Response: In the last paragraph of the introduction (Line 72), we now state that “We hypothesized that participants will incur declines in function and self-reported health following incident chronic disease and that prior function may not be completely regained.” To test this hypothesis, we estimated the slopes of decline after incident disease and the slopes of recovery over subsequent waves. The results of the estimated slopes are given in Tables 2 & 3.

● Response rates are very crucial and if the mean of the response rates for all the interviews is provided, it would be useful- it is just stated that the response rate was over 85%.

Response: We agree that response rates are very crucial. The response rates are complex because we used participants from five HRS cohorts, and every cohort has its own response rate at every interview. We provide more detail on the response rates in the paragraph in Methods under study participants. See Lines 136-139.

● In the study variables section, while defining the conditions, it has been mentioned as heart diseases while later it has been mentioned as ‘heart problems’ in the same resection which is confusing

Response: The HRS uses the term ‘heart problems’ defined as a heart attack, coronary heart disease, angina, congestive heart failure, or other heart problems. We give this definition in the Methods, and now use heart problems consistently unless heart disease is specifically used in a referenced article.

● No mention of deaths during the process or loss of follow-up and how was it accounted for

Response: Participants were kept in analyses for all interviews they attended prior to death. Participants were not dropped from analyses if they missed an interview. Some participants skipped interviews but returned at later exams, so they are not lost to follow-up. We mentioned that 84% of the participants at the baseline exam were known to be alive at the final wave. See Lines 139-142 and Line 182.

● The authors need to specify how can estimation of self-reported measures of muscle strength and mobility can be valid measures? Why were these measures not verified against gold standard?

Response: The measures have been validated against a panel of mental and physical health conditions. Self-reported health validated as a measure of mortality. We now provide the reference on the validation of the measures (see Line 109). Declines in strength, mobility, and self-reported health indicate declines in overall health. We also include references on the validity of self-reported health as a risk indicator of mortality (Lines 112-113).

● It is possible that several covariates associated with multimorbidity, and muscle strength or mobility can confound the association. For example, glucose levels or mental health status. BMI is an important confounder in the chronic conditions mentioned in the study which hasn’t been adjusted for, neither mentioned as a limitation. The authors need to explain the steps taken by authors to minimize the confounding.

Response: We now adjust for body mass index as well as age and sex. We acknowledge in the limitations that diseases may confound the study associations although we did not adjust for the various disease measures available in the HRS at the suggestion of Reviewer #2: See Line 304-306. “the results were controlled for age, sex, and body mass index, but not for health conditions or other social determinants. The results could be biased because of such omitted covariates.”

Apart from confounding, the study methodology suffers from many sources of selection bias (collider bias/ Berkson’s bias etc) and measurement error (differential misclassification of outcome). The authors need to explain why there is no description of minimizing the sources of systematic error.

Response: We added acknowledgement of possible sources of systematic error in the limitations and their possible effects on the study results. Both differential and non-differential misclassification might occur if scores are higher or lower based on participant characteristics, or from error in measurement from using self-report.

The outcomes are based on self-reported health or other factors might introduce systematic errors. Imprecision in measuring the outcomes by self-report could lead to non-differential misclassification.” (Lines 305-310)

The HRS chose instruments with a level of item difficulty useful for participants with both high and low functional status rather than more sophisticated measures. We include this information with a reference in the Methods. (Line 108-109)

Results
● How many years did it take on an average for incident diabetes, stroke, heart problems, and lung problems to lead to decline in self-reported health, muscle strength, and mobility.

Response: We do not have the dates of the incident diseases but know the interviews at which the events were reported. The HRS holds interviews every two years, so that we know that declines occurred within about a two-year interval, i.e., between two interviews.

● Patients developing a second chronic disease experienced additional decline-This is a vague statement-By how much was the decline experienced?

Response: The magnitudes are given in Table 2 in the row labeled “Multiple chronic conditions.” We now give examples in the text to make the results clearer for readers.

● What is the loss to follow up in the study/in the HRS system?

Response: Because participants can skip an interview and return at later interviews, loss to follow-up is not comparable to that of a randomized trial. Participants were included at all interviews they attended and not excluded if they missed interviews. The high response rates of the HRS show that participation remained strong across the decade of follow-up.

● To what extent did the combination of ‘other diseases’ like arthritis, cancer and the chronic diseases and multimorbidities influence the mobility and muscle strength in patients as they were considered for the analysis.

Response: At the suggestion of Reviewer # 2 we excluded the other diseases from the analyses. We now acknowledge this in limitation: “the results were controlled only for age, sex, and body mass index, but not for health conditions or other social determinants. This might affect the estimation of decline in functional limitations and perceptions of health.” Lines 302-305.

● How many patients’ muscle and mobility strength improve with the decline of other diseases like cancer in spite of having diabetes, lung and heart diseases.

Response: The patients are asked “has a doctor ever told you that you have [disease]”. Once diagnosed with a disease participant are treated as having the disease from that interview forward. The HRS does not include data on changes in disease status such as cancer. At the suggestion of Reviewer #2 we no longer include diseases like cancer in our analyses.

● It is unclear why did the authors not present measures of association and have presented only descriptive statistics and self-reported declines and improvements. The manuscript should be revised to include measures of association.

Response: The results in Tables 2, 3, and 4 are from regression models measuring associations of age, sex, body mass index, and time before and after incident chronic disease with muscle strength, mobility, and self-reported health. In the revision we have revised the statistical methods to make the analyses clearer and emphasized what the results represent.

Discussion
● There is no mention of any bias, and how it was addressed, for eg: a respondent’s bias- as muscle strength and mobility among many others were self-reported.

Response: Thanks for the comment. We now add as a limitation that since muscle strength and mobility are self-reported, participants more likely to have declines following incident chronic disease may have under or overestimated their muscle strength or mobility at their baseline exam. We state that the self-reported measures could introduce bias from misclassification and the misclassification could potentially be differential. (Lines 306-308)

● Limitations of the study methodology and causal inference are not mentioned in discussion. The authors need to discuss threats to internal validity in the study and how they tried to minimize or control the sources of systematic error.

Response: We now added acknowledgement of possible sources of confounding and systematic error in the limitations. We chose the HRS because of the high response rates and long-term follow-up retaining the same questions across years of follow-up to minimize sources of error. (Lines 304-311)

**Reviewer #2’s Comments:**
Thank you for considering me to review this manuscript entitled “A longitudinal study of declines and subsequent recovery following incident chronic disease of self-reported health, muscle strength and mobility”. This study aimed to investigate changes in self-reported health, muscle strength and mobility following incident chronic disease. Although the study seems to be relevant from the point of view of public health, I have a few concerns which I have appended below:
Title: Title seems to be a bit confusing. It doesn’t clearly describe the objectives of the study.

Response: We agree. We have simplified the title to: “Relation of incident chronic disease with changes in muscle strength, mobility, and self-reported health: Results from the Health and Retirement Study”

Abstract
Design: Duration of longitudinal follow-up is not mentioned.

Response: We have added the duration of follow-up: “People first interviewed from 1998-2004 were followed across five biannual exams.” See Line 19.

Page 2, Line 6-9: Time points of measurement not mentioned. Each time point should be clearly mentioned in the manuscript.

Response: Time points in the HRS are years of the interviews. The complete list for this study because of the variable first years are 1994, 1996, 1998, 2000, 2002, 2004, 2006, 2008, 2010 and 2012 and 2014.

Now provided on Line 128.

Page 2, Line 17: I doubt if “multi-morbidity” is the right terminology to use

Response: In the revision we use multiple chronic conditions rather than multimorbidity.

Page 3, Line 2: The word “physical declines” is inappropriate. Decline in physical health would be appropriate.

Response: Changed as suggested.

Introduction
Line 20: Remove additional comma after follow-up.

Response: The extra comma is deleted.

Page 4, Line 2: “From before” should be replaced by “even before”
Please state study hypothesis at the end of introduction.

Response: Changed as suggested.

Content of this section appropriate.
Methods
Is there any way by which the sample size of the study could be justified?

Response: The original survey sample size was determined by HRS. The relatively large size of the study population coupled with the extent of the statistical significance and the fairly narrow confidence intervals suggest that the sample size is sufficient.

Page 4, Line 10: Replace “from before” with “since”

Response: Changed as suggested.

Page 5, Line 8-10: It’s a repetition. Can be removed.

Response: We removed the repetition.

Operationalize heart and lung problems for this study.

Response: These are the terms used by the HRS. We give their definitions in Methods under study variables. The definitions are “a heart problem is defined as having a heart attack, coronary heart disease, angina, or congestive heart failure; a lung problem as chronic lung disease such as chronic bronchitis or emphysema but except asthma; and stroke as stroke or transient ischemic attack.”

Page 6, Line 8: Why skin cancer is kept as an exception?

Response: Skin cancer is no longer a criterion, but most skin cancers are benign, which is why skin cancer was kept as an exception. Melanoma accounts for only about **1%** of skin cancers but causes a large majority of skin cancer deaths.

Line 9: What do you mean by “nervous problems”. It’s not a scientific terminology.

Response: The variable “nervous problems” is not used in the revision.

It is strange that no validated questionnaire was used for assessing self-reported health rather a simple Likert scale was utilized.

Response: The HRS chose measures suitable for participants with both high and low functional statuses. The HRS considered subjective impressions important and wanted measures that could be used with large cohorts. The Likert scale for self-reported health has been widely used in epidemiologic studies and validated as a predictor of mortality. We provide with references to self-reported health in the Methods. See Lines 112-113.

Similarly, no validated test was used for assessing muscle strength. The tasks utilized to assess muscle strength seems to measure mobility and mobility and strength are two different domains.

Response: Muscle strength and mobility were validated against eight health conditions and found to have strong associations. The reference is provided in the manuscript (reference 17). Muscle strength and mobility are considered to summarize overall health. It is an advantage that the HRS used the same instruments at every interview.

Statistical analysis
Adjusting age as a covariate in this analysis makes sense. However, covariates such as arthritis, cancer and psychiatric problems are not justified. Why only these diseases were considered covariates and not any other disease?

Response: In the revision we adjust for age, sex, and BMI categories (normal, overweight, & obese), but not for chronic diseases. We agree that the HRS collected limited data on chronic disease and used broad self-reported categories. We add to limitations that health outcomes not controlled in our analyses may have affected the declines.

Couldn’t understand this section (written in a complex manner). Try to simplify.

Response: We have re-written the statistical analysis section to make it easier to understand.

Discussion
Page 11, Line 19-20: “These results show the risk facing----older adults”- re-phrase

Response: Rephrased in revision in the sentence of second paragraph of Results. See Lines 255-256.

Page 12, Line 14: “Our” is superscripted. Please correct.

Response: Corrected.

Page 13, Line 7: “Limitations”-it’s repeated

Response: The repeat is deleted.

Page 8, Line 12-17: There is minimal discussion on main outcome variables of the study.

Response: We have expanded the discussion of the outcome variables in the first paragraphs of the Discussion.

Page 14, Line 1: Should be “variable times”

Response: Variables times changes to variable times.

Page 15, Line 17: “healthy older adults”-correct
Overall, this section is vaguely written. There is minimal discussion in support of main objective of this study which should be adequately incorporated.

Response: We have expanded the discussion both to give greater emphasis on the primary outcomes and to provide the public health implications of the results (beginning and end of the discussion.

Conclusion

Conclusion of the study is inadequately written. Authors need to strongly state the application of the study on societal level. Clear application to public health should be stated.

Response: We have strengthened the final paragraph emphasizing the public health and societal applications of the findings.
