# Supplementary material for: Relation of incident chronic disease with changes in muscle function, mobility, and self-reported health: Results from the Health and Retirement Study
Source: PLOS Glob Public Health. 2022 Sep 8;2(9):e0000283. doi: 10.1371/journal.pgph.0000283 (PMC10021154; doi:10.1371/journal.pgph.0000283)
Supplement: Attachment — Submitted filename: PGPH responses to reviewers 7 12.docx [file pgph.0000283.s002.docx]

**Response to Reviewers’ Comment on PGPH-D-21-00399**

● Reviewer #1: Thanks for addressing all the comments.

Response: We appreciate the review.

**Reviewer #2’s Comments:**

Reviewer #2: Dear Authors,
I am still not satisfied with the author's responses on use of **non-validated questionnaires for measuring self-reported health** which is an important variable in this study despite existence of several validated tools.

Response: The question we use is the first question from the SF-36 questionnaire that is widely used to assess physical and mental health. The question is validated against mortality, considered by some to be the best objective measure of health of an individual. We add a recent reference that examines the association with mortality in some detail (reference 21. We agree, as a single question, the question we use lacks the multidimensionality of some other self-reported health questionnaires. We include this information as a limitation.

Though muscle strength and mobility are validated against health conditions and are strongly associated but still usage of a tool meant to measure mobility is not justified for strength. It’s better not to name the variable as strength.

Response: In the revision were use the term ‘muscle function’ in place of muscle strength. This term reflects the three functional tasks asked about (walking, climbing, and bathing).

As authors said that they have replaced the previous covariates with sex and BMI now. Are there any changes in the results with new co-variates? Removing the clinical conditions as co-variates also doesn't solves the matter as they may influence the relationship between dependent and independent variables. Rather the utilization of relevant diseases as covariates should be justified. You may keep sex and BMI as covariates as well.

Response: We have redone the analyses for our tables 2 & 3. Adding chronic diseases to the models led to only limited changes in the estimated coefficients and the statistical significance of the results in the tables did not change. In the methods we now explain why the chronic conditions are included (lines 126 -128). The new tables adjusted for chronic conditions are included in the revised manuscript.

Discussion of the manuscript can be further improved.

Response: We have revised and extended the Discussion section.

.
